# Remote patient monitoring at home using ambient sensors: a systematic review

**DOI:** 10.1093/eurpub/ckac130.065

**Published:** 2022-10-25

**Authors:** A Alboksmaty, N Solomons, S Gul, AL Neves, P Aylin

**Affiliations:** 1 Department of Primary Care and Public Health, Imperial College London, London, UK; 2 School of Public Health, Imperial College London, London, UK; 3 PSTRC, Imperial College London, London, UK

## Abstract

**Background:**

The world population is ageing, and their health needs imply substantial demands on health systems. Remote Patient Monitoring (RPM) may help elderly patients live independently in their homes for longer. The essence of RPM is the continuity of use, which is challenging for wearable devices and patient-led technologies. Unobtrusive (ambient) sensors could be an innovative solution, such as motion detectors and similar technologies. This study aims to review the evidence on the effect of ambient sensors on healthcare use by the elderly.

**Methods:**

This is a systematic review for narrative synthesis, searching five databases, Medline, Embase, CINHAL, Scopus and Web of Science, on 21 Feb 2022 without setting a lower time limit. No restrictions on the design of studies were applied. A meta-analysis was not feasible due to the heterogenicity of the studies.

**Results:**

Out of 5,653 search results, 180 studies were subjected to full-text review, of which 6 studies were included in the final synthesis. All the included studies were conducted in the USA. Four studies assessed the technology's cost-efficiency, while only one reported significant cost savings. One study reported a significant reduction in hospital days and visits to a physician among the users. Using ambient sensors was associated with an increased length of stay in facilities where the elderly can live independently, including at home. The impact on the number of hospitalisations or emergency room visits was unclear.

**Conclusions:**

Our review identified limited evidence on the effect of ambient sensors on healthcare use by the elderly. The potential has been demonstrated for ambient sensor technologies to result in cost savings; however, further research is needed to assess the impact on health outcomes.

**Key messages:**

- Ambient sensors can be effectively used to remotely monitor the health of eldelry people at home.
- Further research is needed to assess the cost effectiveness of using ambient sensor technologies in remote patient monitoring.

